# Reverse Genetics Assembly of Newcastle Disease Virus Genome Template Using Asis-Sal-Pac BioBrick Strategy

**DOI:** 10.1186/s12575-020-00119-3

**Published:** 2020-05-01

**Authors:** Amin Tavassoli, Safoura Soleymani, Alireza Haghparast, Gholamreza Hashemi Tabar, Mohammad Reza Bassami, Hesam Dehghani

**Affiliations:** 1 Division of Biotechnology, Faculty of Veterinary Medicine, Ferdowsi University of Mashhad, Azadi Square, Mashhad, Iran; 2 Immunology Section, Department of Pathobiology , Faculty of Veterinary Medicine, Ferdowsi University of Mashhad, Mashhad, Iran; 3 Department of Basic Sciences, Faculty of Veterinary Medicine, Ferdowsi University of Mashhad, Mashhad, Iran; 4 Stem Cell Biology and Regenerative Medicine Research Group, Research Institute of Biotechnology, Ferdowsi University of Mashhad, Azadi Square, Mashhad, Iran

**Keywords:** Newcastle disease virus, Asis-Sal-Pac-BioBrick strategy, Synthetic biology, Reverse genetics technology

## Abstract

**Background:**

The BioBrick construction as an approach in synthetic biology provides the ability to assemble various gene fragments. To date, different BioBrick strategies have been exploited for assembly and cloning of a variety of gene fragments. We present a new BioBrick strategy, here referred as Asis-Sal-Pac BioBrick, which we used for the assembly of NDV as a candidate for single-stranded non-segmented, negative-sense RNA genome viruses.

**Results:**

In the present study, we isolated three NDVs from clinical samples which were classified into the VIId genotype based on their pathogenicity and phylogenetic analyses. Then, SalI, AsisI, and PacI enzymes were used to design and develop a novel BioBrick strategy, which enabled us to assemble the NDV genome, adopting the “rule of six”. In this method, in each assembly step, the restriction sites in the newly formed destination plasmid are reproduced, which will be used for the next insertion. In this study using two overlapping PCRs, the cleavage site of the F gene was also modified from ^112^RRQKRF^117^to ^112^GRQGRL^117^ in order to generate the attenuated recombinant NDV. Finally, in order to construct the recombinant NDV viruses, the plasmids harboring the assembled full-length genome of the NDV and the helper plasmids were co-transfected into T7-BHK cells. The rescue of the recombinant NDVwas confirmed by RT-PCR and HA tests.

**Conclusions:**

These findings suggest that the combination of reverse genetic technology and BioBrick assembly have the potential to be applied for the development of novel vaccine candidates. This promising strategy provides an effective and reliable approach to make genotype-matched vaccines against specific NDV strains or any other virus.

## Background

An important goal in synthetic biology is to assemble various gene fragments in order to make the process of engineered gene expression more efficient and more reliable [1]. The BioBrick assembly standard processes provide the means to easily put together the DNA sequences of defined structure and function. BioBricks as a standard assembly process was described by Knight and coworkers in 2002 [2]. In fact, the assembly of two parts together will preserve the standard prefix and suffix sequences (including restriction enzymes) for future assemblies. Thus, the newly formed composite can again be recombined with any other BioBrick part in the next round of assembly, using the same restriction enzymes. The majority of the BioBrick standard parts submitted to the parts registry are designed for the work with *Escherichia coli* [3, 4]. An assembly technique to put together a larger number of gene fragments, or produced from the genome of other microorganisms, especially RNA viruses, have not been developed.

Newcastle disease (ND) is one of the most severe respiratory and neurological diseases of poultry which is caused by the Newcastle disease virus (NDV) [5, 6]. It is able to infect over 240 species of birds and can be transmitted and spread by inhalation or ingestion. Infected birds shed this virus in feces, as well as in the respiratory secretions [7]. Based on the severity of the disease in chickens, the causing NDV strains have been classified into lentogenic, mesogenic, and velogenic pathotypes. Lentogenic NDV produces the disease with mild respiratory or enteric symptoms [8–10]. Mesogenic NDV strains show intermediate virulence and can cause respiratory infection with histological lesions (in less than 10% of cases). However, the velogenic strains can cause significant respiratory, neurological, and digestive tract pathologies with high mortality rates up to 100% [11, 12].

NDV belongs to the genus Avulavirus, family Paramyxoviridae. NDV is an enveloped virus with a single-stranded negative-sense RNA genome, containing more than 15 k nucleotides that encodes six main proteins. These sequences in 3′ to 5′ direction encode the nucleocapsid protein (NP), phosphoprotein (P), matrix protein (M), fusion protein (F), hemagglutinin-neuraminidase (HN), and RNA-dependent RNA polymerase (L), separated by non-transcribed intergenic (IG) sequences named gene-end (GE), and gene-start (GS) [13–15]. Fusion protein and hemagglutinin-neuraminidase cooperate in the invasion of host cells and also determine viral virulence [16, 17]. The molecular determinant of NDV pathogenicity is the amino acid sequence of the F protein cleavage site and its amino acid makeup. The F protein is synthesized as inactive precursor F0, which is cleaved by the host proteases into two subunits, F1 and F2, to activate its fusion activity. The virulent NDV viruses have the characteristic sequence of 112R/K-R-Q-K/R-R116 at the C terminus of the F2 subunit and phenylalanine at residue 117 at the N terminus of the F1 subunit. However, viruses with low virulence have the characteristic sequence of 112 G/E-K/R-QG/E-R116 at the C terminus of the F2 subunit and leucine at residue 117 at the N terminus of the F1 subunit [18–21].

NDVs have been grouped into two major classes (Class I and II), and into several genotypes and sub-genotypes [22]. Based on phylogenetic analysis of the fusion (F) protein gene, the class I NDV has been divided into nine genotypes, while class II comprises 16 genotypes. Class II NDVs comprise the majority of virulent and some avirulent NDV strains. Almost all strains in genotype VII of class II NDVs are velogenic, and according to amino acid substitutions can be further divided into five (VIIa–e) subgenotypes [23–26]. The genotype VII NDV has become the most important genotype circulating in Iran. The intensive vaccination program has been implemented in Iran, but ND is endemic, and NDV VII strain outbreaks occasionally occur in various parts of the country [27–30].

The conventional vaccines against ND, such as La-Sota and B1 are widely used live-attenuated vaccines that comprise strains which were isolated some 70 years ago to control the disease in poultry and belong to classical genotype II [31, 32]. Low efficacy of these vaccines has been the cause of a major shift in the types of prevalent NDV strains that have been identified in poultry. On the other hand, researchers have shown that NDV vaccines from viruses, genetically closer to the outbreak viruses, provide better ND control by reducing virus shedding from infected birds [33–36].

Reverse genetics techniques for non-segmented, single-strand negative-sense RNA viruses, was first developed for rabies virus by Conzelmann [37]. In this system, co-transfection of a plasmid expressing full-length anti-genomic RNA together with helper plasmids encoding viral proteins, forms the ribonucleoprotein (RNP) complex, under the control of the phage T7 RNA polymerase promoter, resulting in the recovery of recombinant viruses [36, 38, 39]. In usual reverse genetics techniques, the endogenous restriction enzymes are used to assemble cDNA-encoded full-length antigenomic RNA [39–41]. In fact, all experiments to construct a recombinant Newcastle disease virus using reverse genetics technique have been carried out with endogenous restriction sites within the NDV’s sequences or using restriction sites introduced by PCR in the downstream untranslated region (UTR) of the genes [32, 38, 39, 42]. The final goal after this process would be to produce genetically modified NDVs in which the virulent F protein cleavage site motif has been modified to an avirulent motif. Thus, an efficient system for reconstitution of new strains and targeted manipulation of the virulence genes is urgently needed in order to speed up the achievement of new strains which are used as vaccine seeds.

In this study, we present a novel method, Asis-Sal-Pac BioBrick, for assembly of individual genes of the large genome of NDV into a full-length genome. This method enabled us to use the same restriction enzymes (AsisI, SalI, and PacI) in sequential reactions [1, 43, 44]. The successful use of this novel assembly procedure to engineer the whole genome of NDV confirms that this methodology could be used for assembly of the genome in many other viruses.

## Results

### Identification and Characterization of the Local Newcastle Disease Virus Velogenic Strains

The three isolates of NDV reported in this study were collected from clinical specimens of Northeastern Iran during 2014–2016. The pathogenicity analyses of the all three isolated NDVs were performed by two tests of the intracerebral pathogenicity index (ICPI) in 1-day-old SPF chickens, and the mean death time (MDT) in 10-day-old embryonated SPF chicken eggs, according to international OIE standards. The isolated strains were velogenic on the basis of both the ICPI (> 1.8; Supplementary Table S1) and MDT (< 53-h; Supplementary Table S2) values, as previously described by Alexander (1989). According to these pathogenicity indices, the ICPI more than 1.2 and MDT less than 60-h could be categorized as velogenic.

**Fig. 1:**
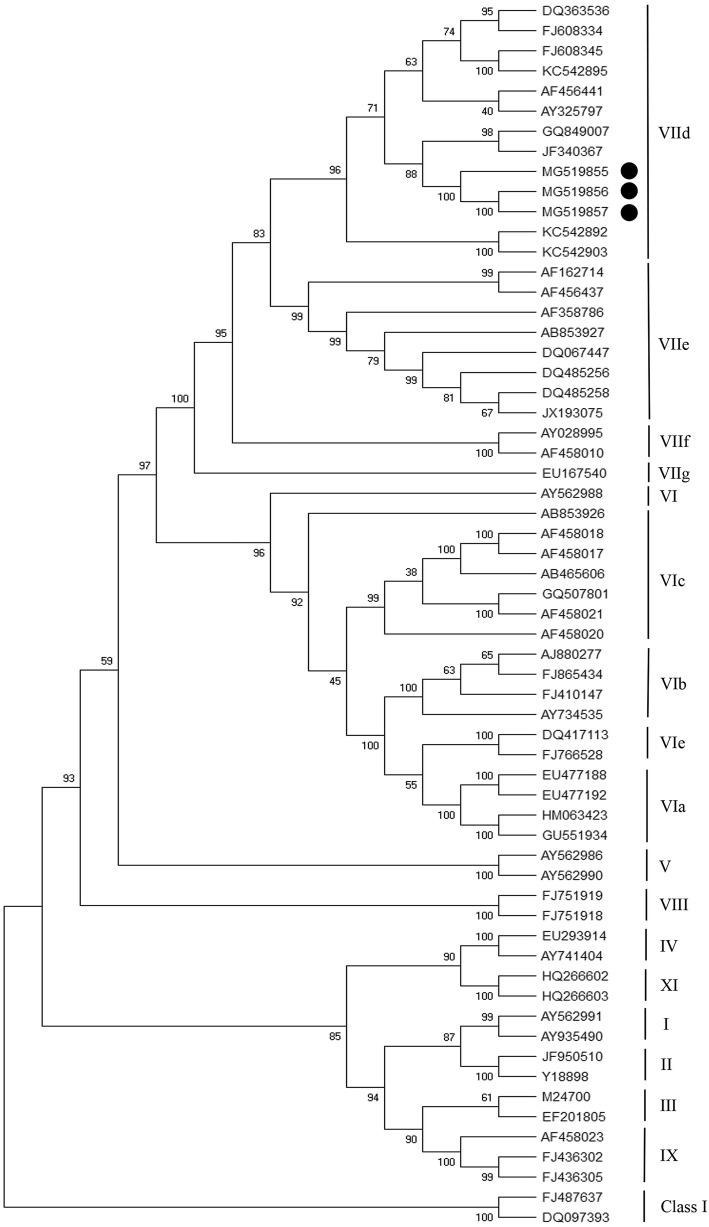
Phylogenetic tree of NDV strains based on the nucleotide sequence of the F gene (entire ORF; 1 to 1662 nucleotide). The sequences of the F gene from other regions, published in GenBank, are listed in the Supplementary Table S1. The provisional designations of the genotypes are indicated on the right. The phylogram was generated by the neighbor-joining method with 500 bootstrap replicates using MEGA X software. The sequences of the isolated strains in this study are marked with black dots

To assess the F0 protein proteolytic cleavage site motifs (residues 112 to 117), the sequences of the isolated Iranian NDVs were determined to be ^112^RRQKRF^117^, the signature sequence of the F cleavage site motif in the velogenic strains. The nucleotide sequences of the isolated NDVs were registered in GenBank and are available with the allocated accession numbers (Table 1). Phylogenetic analyses were performed on the complete coding region of F gene sequence (nucleotide 1 to 1662) from the NDV strains (Supplementary Table S3) using MEGAX (MEGA, version X) software with statistical analysis based on 500 bootstrap replicates (Fig. 1). Based on the phylogenetic tree, the Iranian NDVs isolated in this study were classified in subgenotype VIId of genotype VII, class II.

**Table 1:** Pathogenicity indices and fusion protein cleavage site sequences of 3 Iranian NDV isolates

	Isolate 1	Isolate 2	Isolate 3
Pathogenicity index: MDT	52.27	43.5	50
Pathogenicity index: ICPI	1.80	1.85	1.84
Cleavage site sequences of the F protein	^112^RRQKRF^117^	^112^RRQKRF^117^	^112^RRQKRF^117^
GenBank Acc. No.	MG519855	MG519856	MG519857

### Assembly of Recombinant Newcastle Disease Virus Whole Genome Using Asis-Sal-Pac BioBrick Strategy

**Fig. 2:**
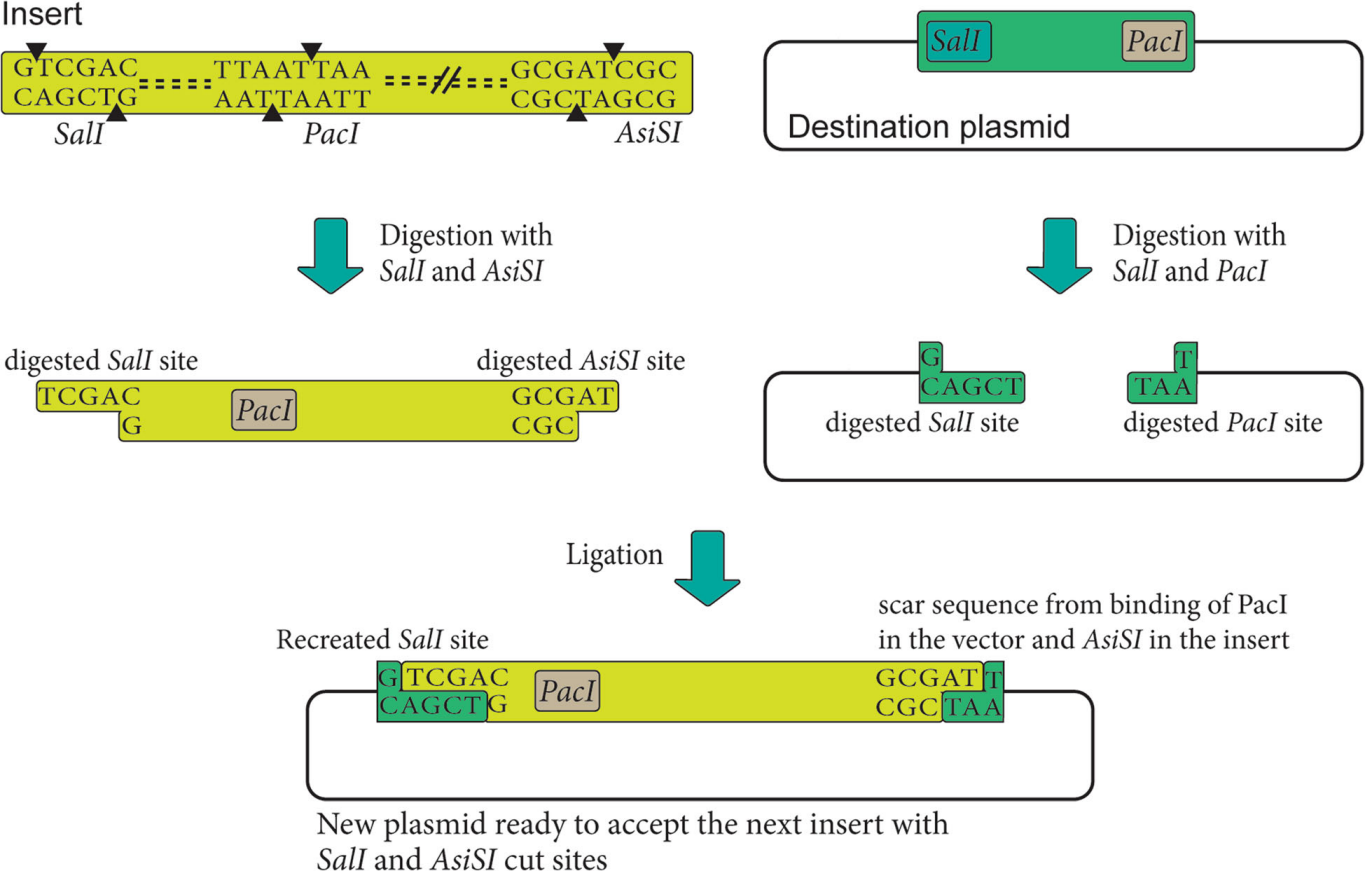
Schematic basis of Asis-Sal-Pac BioBrick strategy. To perform this assembly, a destination plasmid with two adjacent restriction sites of SalI and PacI is needed. The inserts with two restriction sites of SalI and PacI at the 5′-end, and an AsisI restriction site at the 3′-end will be used. Each assembly step results in the ligation of SalI sites from the plasmid and insert, re-creating a new SalI recognition site at the 5′-side of the insert. However, at the 3′-side of the insert the AsisI site after digestion will be compatible with PacI site in the destination plasmid, forming a scar sequence after ligation. The new plasmid with recreated SalI and the PacI site (sequence of the previous insert which is currently part of the vector sequence) will be ready to accept the next insert with SalI, PacI, and AsiSI sites in the next assembly step

In this study, we have designed and developed a novel BioBrick strategy that uses three restriction sites of SalI, AsisI, and PacI (Fig. 2). This strategy enabled us to construct the complete genome of NDV in sequential steps. The previously published BioBrick strategies have been employed to assemble limited size DNA contigs. However, we have been able to assemble a genome of 15,192 bp, using this strategy adopting the “rule of six” for NDV genome. In this strategy, in each assembly step, a new SalI restriction site will be created in the plasmid. However, the AsisI site at the 3′-side of the insert will be compatible with the PacI site in the destination plasmid, forming a scar sequence after ligation. Thus, the new plasmid after the first assembly will have a recreated SalI and an adjacent PacI site (belonging to the sequence of the insert before ligation). Since this plasmid has lost its previous PacI site, the next restriction enzyme digestion could not cut the previously assembled fragment. Hence, this plasmid will be ready to accept the next insert with SalI, PacI, and AsiSI sites in the next assembly step (Fig. 2). This feature of Asis-Sal-Pac BioBrick strategy could be used to assemble any number of fragments as long as the destination plasmid can accommodate.

**Fig. 3:**
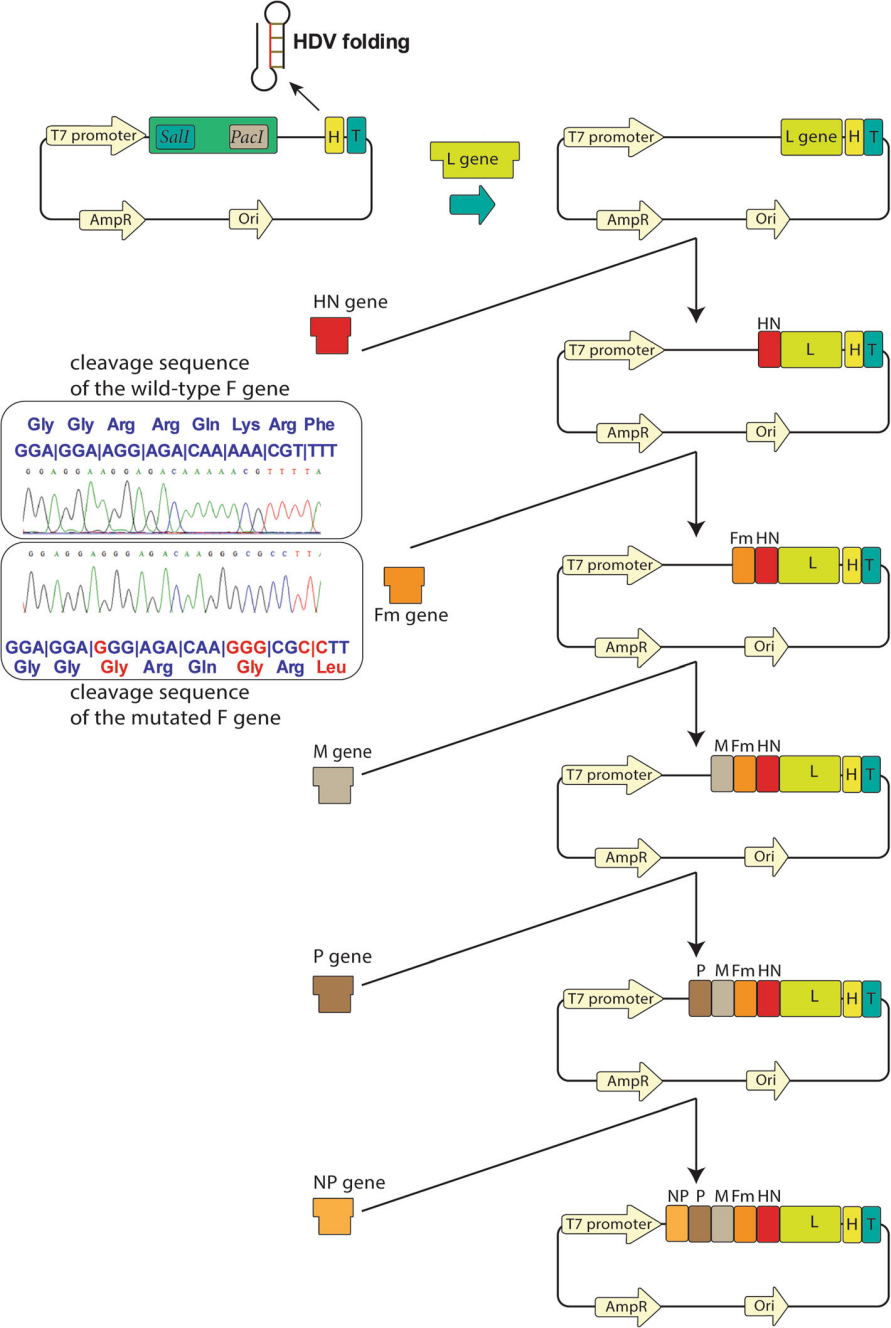
Schematic overview for the generation of the complete genome of recombinant Newcastle disease virus (rNDV) using Asis-Sal-Pac BioBrick strategy. The Iranian NDV isolates were subjected to RT-PCR. Six subgenomic fragments of L, HN, Fmut, M, P, and NP were cloned sequentially into the Asis-Sal-Pac-BioBrick vector, flanked on the upstream side by a T7 RNA polymerase promoter (T7 promoter) sequence and, on the downstream side by the hepatitis delta virus ribozyme sequence (H) followed by a T7 terminator sequence (T). To generate the attenuated recombinant NDV, we introduced and confirmed (by Sanger sequencing) mutations in the cleavage sequence of the wild-type F gene (here named Fm). The assembled genome on the vector was designed in accordance with the “rule of six” for NDV

To construct the full-length anti-genome of the Iranian NDV isolates, cDNA clones expressing six open reading frames of the NDV transcript were generated and assembled using the Asis-Sal-Pac-BioBrick strategy (Figs. 2 and 3). In this study, six cDNA segments that were synthesized by RT-PCR from the viral genomic RNA were sequentially inserted into the Asis-Sal-Pac BioBrick destination plasmid (Fig. 3). We designed the destination plasmid and following insertions in accordance with the “rule of six” for NDV genome. In addition, to generate the attenuated recombinant NDV, the cleavage site of the F gene was replaced (using two overlapping PCRs) with a cleavage site that is common in lentogenic strains, and its sequence was modified from^112^RRQKRF^117^ to ^112^GRQGRI^117^ (Fig. 3).

### Rescue of Genetically Modified NDV

**Fig. 4:**
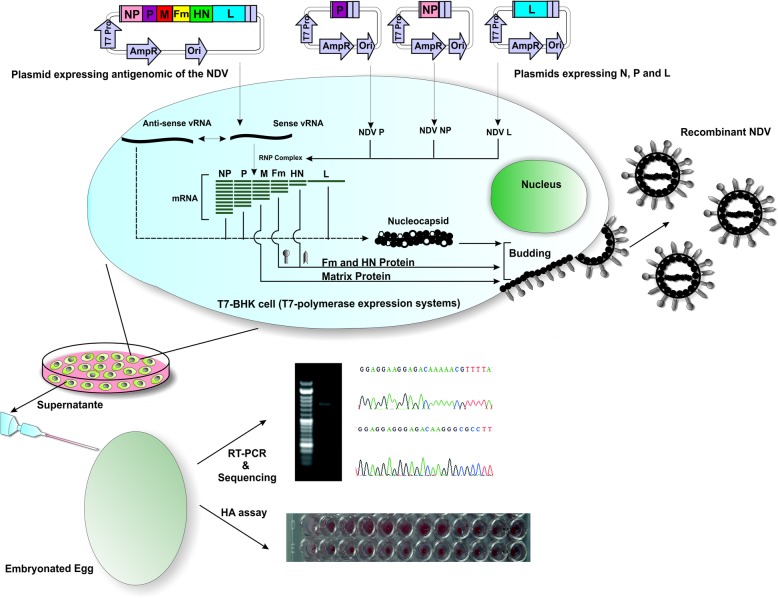
Schematic representation of the reverse genetics technique for the rescue of recombinant Newcastle Disease Virus. Plasmids expressing the whole anti-genome, and the helper genes (NP, P, and L), constructed using the Asis-Sal-Pac BioBrick strategy, were used to transfect the T7-BHK monolayer cells. The amounts of different plasmids for transfection were adjusted in a way to express genes with different mRNA levels. Reconstituted RNPs in the cytoplasm, with the transcribed antigenome RNA, are used to assemble the new viral genomes and to produce new viral particles, which are released via budding from the infected cells into the supernatant. Forty-eight hours post-infection, the supernatant was harvested and inoculated into 10 days-old embryonated eggs. After 5 days, the allantoic fluid of the inoculated eggs was analyzed for the presence of viral hemagglutinin by HA assay, and for the presence of viral RNA by RT-PCR and Sanger sequencing. –vRNA: anti-sense viral RNA; +vRNA: sense viral RNA; F: fusion protein; HN: hemagglutinin-neuraminidase, M: matrix; L: large polymerase protein; P: phosphoprotein; NP: nucleoprotein

To generate the recombinant NDV viruses, the plasmids harboring the assembled genome of NDV and helper genes (N, P, and L) were used to transfect T7-BHK as previously described [32, 39]. Analysis of the culture medium by inoculation of the allantoic cavities of 10 day-old embryonated eggs, and the following HA and RT-PCR analyses confirmed that we have been able to successfully generate the recombinant NDV using Asis-Sal-Pac BioBrick strategy. The allantoic fluids were found to be HA-positive for 8 log^2^ dilutions. The presence of the mutated F protein was confirmed by sequencing of the PCR product, acquired from RT-PCR of the allantoic fluid injected with the recombinant virus (Fig. 4).

## Discussion

Previous investigations in synthetic biology have shown that the assembly of large and manifold DNA fragments is a time consuming, expensive, and often unpredictable process, both in research laboratories and at gene synthesis companies [1, 43]. Among different strategies available for DNA construction, the BioBrick assembly is one of the important techniques to build long DNA sequences [43, 45, 46]. To date, different assembly standards have been designed and optimized for different targets. Here, we report that a new version of the BioBrick assembly using AsisI, SalI, and PacI restriction enzymes (the Asis-Sal-Pac BioBrick strategy) can be applied for whole-genome assembly of NDV, a virus with single-stranded and negative-sense RNA genome.

A member of genotype VII has been the main cause of recent outbreaks of ND in Asia, and especially Iran [27, 29]. This indicates that the use of vaccines made from strains different from pathogenic local NDVs might render them inefficient. It has been shown that the genotype-matched strain vaccines have acted far better in the prevention of infection than conventional vaccines [36]. Thus, various types of reverse genetics approaches and genome assembly processes for the construction of recombinant NDV have been established [32, 36, 39]. A whole-genome assembly of NDV using Asis-Sal-Pac BioBrick strategy leads to fast construction of the whole genome of viruses that are similar to circulating NDVs and are able to present the majority of immunogenic epitopes lacking the same pathogenicity.

It is established that in response to immune pressure, NDVs shedding leads to returning these viruses to velogenic strains. The reason for this occurrence is a mutation in the viral population in vaccinated chickens [31]. In our developed standard method, there is the possibility to generate a new recombinant NDV vaccine during the outbreak using the causing genotype in a fast and reliable manner.

## Conclusions

In conclusion, we have designed and developed a new BioBrick strategy that enabled us to clone and assemble the genome of circulating velogenic NDVs without any deletion or addition of internal recognition restriction sites, with intact ORFs. This study shows that the important barrier of using a large number of enzymes that have to be cutter for the genome of the virus and non-cutter for the vector can be easily removed by using the Biobrick strategy. In fact, our method reduces the number of restriction enzymes to a minimum and to common enzymes. The BioBrick strategy reported in this study can be used to construct full-length genome of other virus members of the Paramyxoviridae family with the non-segmented and single-stranded genomes, such as Mumps rubulavirus, Measles virus, and Henipavirus.

## Methods

### Cells and Viruses

The three isolates reported in this study were collected from clinical specimens of Northeastern Iran during 2014–2016. Isolates were inoculated into 10-day-old embryonated SPF eggs (Razi Vaccine and Serum Research Institute, Karaj, Iran) and incubated at 37 °C. The infected allantoic fluids were harvested 2 days post-inoculation and analyzed for the existence of NDV by hemagglutination assay (HA) and stored at − 80 °C until use. For HA assay, 0.025 ml phosphate buffer saline (PBS) was added to each well on a microtiter plate (with U-shaped wells). Next, 0.025 ml of harvested allantoic fluid containing NDV was added to the first well. The mixture in the first well was serially diluted by transferring 0.025 ml volume from one well to the next. Then, 0.025 ml of 1% (diluted with PBS) chicken red blood cells was added to each well. The red blood cells-added plates were incubated at room temperature for 30 min and were visually monitored for the formation of aggregates.

The velogenic potential of each isolate was evaluated using standard assays of intracerebral pathogenicity index (ICPI) and mean death time (MDT). All tests were performed according to international OIE standards [47]. The isolates were confirmed by RT-PCR. NDV isolates were plaque-purified on primary chicken embryo fibroblasts (CEFs) in three rounds as previously described [48, 49].

The Primary chicken embryo fibroblasts (CEFs) were derived from 10 days-old embryonated specific-pathogen-free (SPF) chicken eggs [50] and were grown in Dulbecco’s minimal essential medium (DMEM) with 10% fetal bovine serum (FBS). The T7-BHK cell line that expresses T7 RNA polymerase was purchased from the Pasture Institute of Iran and was grown in α-MEM supplemented with 5% FBS [51].

### RNA Isolation, Sequencing, and Phylogenetic Analysis

The allantoic fluids including the viral RNA of the NDV were subjected to RNA isolation using Total RNA purification kit (Jena Bioscience Co., Germany) according to the manufacturer’s instructions. Afterward, the extracted viral RNA was transcribed to cDNA with random hexamer primers using AcuuPower RT PreMix (Bioneer Co., Korea). Briefly, a total of 250 ng viral RNA was added to each AccuPower CycleScript RT PreMix (dN_6_) tube, subsequently filled up to 20 μL with DEPC water. The lyophilized premix pellet was dissolved by taping the tube and vortexing. cDNA was synthesized in 12 cycles of 20 °C for 30 S, 43 °C for 4 min, and 55 °C for 30 S. At the end, the RT enzyme was heat-inactivated at 95 °C for 5 min. In order to obtain the complete coding sequence of the F gene, the PCR amplification was performed using Phusion High-Fidelity PCR Kit (Thermo Fisher Scientific Inc., USA) with the following thermocycling conditions; 98 °C for 30 S, followed by 33 cycles (98 °C for 10 S, 60 °C for 20 S, and 72 °C for 20 S/kb) and a final extension step of 7 min at 72 °C. The PCR reaction components were 2 μL cDNA, 1 μL forward and reverse primers (10 μM), 0.4 μL dNTPs (10 μM), 4 μL Phusion HF buffer (5X), DMSO 0.6 μL, 0.2 μL Phusion DNA Polymerase, and dH_2_O to 20 μL final volume. PCR primers used in this study were designed according to the consensus sequences of F genes from the GenBank database of the National Center for Biotechnology Information and using AlleleID software (version 7). The primers are listed in Supplementary Table S4.

To assess the genetic relatedness of the NDV strains, PCR products were electrophoresed with 1% agarose gel and purified using AccuPrep® Gel Purification Kit (Bioneer, Korea). Then, the recovered PCR products were sequenced in both directions by Sanger sequencing (Macrogen Inc., South Korea). The assembly and editing of sequences were performed using BioEdit and phylogenetic analysis was performed on the open reading frame of the F gene. Since the F gene is the main determinant gene for virulence of NDV, it was used for making a phylogenetic tree. The GenBank accession numbers for the three isolates sequenced in this study are MG519855, MG519856, and MG519857. The confirmation of genotypes and sub-genotypes of the isolates were conducted by the phylogenetic tree and the evolutionary distances between different groups using the neighbor-joining method (Kimura 2-parameter) of MEGA software (version X) by a comparison of the nucleotide sequences of the entire ORF of the F gene from 1 to 1662 nucleotides. The reliability of the tree was evaluated by bootstrap analysis with 500 replicates.

### Construction of the Specific Plasmid for Asis-Sal-Pac BioBrick Strategy

The plasmid dedicated for transcription of the full-length NDV anti-genome and helper plasmids (encoding NP, P and L proteins) using Asis-Sal-Pac BioBrick strategy was created as follows. First, pGH Vector was digested by EcoRI and XhoI, and a 52-nucleotide linker containing AsisI, SalI and PacI restriction sites was introduced to make the vector pGH-Asis-Sal-Pac-BioBrick. The sequence of the ribozyme of hepatitis delta virus (HdvRz; pJL 89 vector from AddGene) was synthesized by annealing of a pair of oligonucleotides which was ligated into the pGH-Asis-Sal-Pac at SalI and PacI sites to make pGH-HdvRz-Asis-Sal-Pac BioBrick vector. Next, the T7 termination sequence was amplified by PCR from PET 21b vector and was ligated into the pGH-HdvRz-Asis-Sal-Pac BioBrick vector at SalI and PacI sites located just after HdvRz sequence. The resulting vector, pGH-HdvRz-T7term-Asis-Sal-Pac-BioBrick, was used for the assembly of the NDV genome (from the MG519856 isolate). The helper plasmids encode L, P, and NP genes. The products of these gene form the RNP complex, a structure that serves as a template for the viral RNA polymerase.

### Direct Mutagenesis of the F Gene

The sequence encoding the protease cleavage site of the fusion protein (F) was modified by PCR direct mutagenesis. The mutagenesis was performed using two overlapping PCR fragments. The mutagenesis primers were designed based on the nucleotide sequence of the avirulent NDV strains. The first PCR fragment was generated using the NDV-Fgene-F and NDV-FRmut primers. The second PCR fragment was generated using NDV-FFmut and NDV-Fgene-R primers. The nucleotides shown in Italic in Supplementary Table S4 are modifications introduced in the primers to change the amino acid sequence of the cleavage site from GRRQKR↓F to the consensus sequence of avirulent NDV strains (GGRQGR↓L). The two overlapping PCR products were joined in a second PCR by using NDV-Fgene-F and NDV-Fgene-R primers. The sequence alterations in the F gene were introduced in a way that did not break the “rule-of-six” [5]. The resulting F gene mutant fragment encoding the avirulent protease cleavage site was introduced into to the pGH-HdvRz-T7term-Asis-Sal-Pac BioBrick vector.

### Construction of Full-Length NDV Using Asis-Sal-Pac BioBrick Strategy

Cloning procedures for the assembly of the entire 15,192 bp genome of the NDV were performed using Asis-Sal-Pac BioBrick strategy. Whole viral genome was generated by reverse transcription-polymerase chain reaction (RT-PCR) in eight fragments. L gene, due to its considerable length, was divided into three parts. The first part of the L gene had two restriction sites of SalI and BsrGI at the 5′-end, and an AsisI restriction site at the 3′-end. The second part of the L gene had two restriction sites of SalI and NheI at the 5′-end, and a BsrGI restriction site at the 3′-end. Eventually, in the third part of the L gene, there were two restriction sites of SalI and PacI at the 5′-end, and a NheI restriction site at the 3′-end. For the remaining five genes of the NDV, each fragment had two restriction sites of SalI and PacI at the 5′-end, and an AsisI restriction site at the 3′-end. Assembly of the whole genome was in accordance with the “rule of six”. In the NDV genome, each NP subunit is in contact with exactly six nucleotides. This arrangement is required for efficient replication (Schirrmacher, 2009). The plasmids expressing NP, P, and L genes as helper plasmids of the NDV were generated by cloning the open reading frames of NP, P and L genes into the specific plasmid of the Asis-Sal-Pac BioBrick.

### Recovery of Recombinant NDVs

T7-BHK cells grown to 60% confluency in 6 cm-diameter dishes were washed twice with Opti-MEM (Invitrogen, CA). For adjusting the level of mRNA expression, we transfected cells with a total of 5 μg plasmids including pNDV, pNP, pP, pL at the ratio of 1:0.5:0.25:0.1 using Lipofectamine 3000 (Thermo Fisher Scientific Inc., USA) added to 2 ml of Opti-MEM. Transfection was performed according to the manufacturer’s instructions. After 6 h, the medium was replaced with 6 ml of Opti-MEM. On the second day after transfection, the supernatant was harvested and 0.2 ml of the supernatant was inoculated into the 10 days-old embryonated chicken eggs. To identify the rescued virus, the allantoic fluid was examined using the HA test, RT-PCR to amplify the F gene, and its sequencing to confirm the presence of the mutation introduced into the cleavage site.

## Supplementary information

**Additional file 1: Table S1.** Intracerebral pathogenicity index (ICPI) for three isolated NDVs in 1 day-old chickens.

**Additional file 2: Table S2.** Mean death time (MDT) for three isolated NDVs in SPF embryonated eggs.

**Additional file 3: Table S3.** The NDV strains used in the analysis of phylogenetic tree.

**Additional file 4: Table S4.** List of primers for identification, amplification, mutagenesis, annealing, and sequencing.

## Data Availability

The data sets used and/or analysed during the current study are available from the corresponding author on reasonable request.

## Abbreviations

ND: Newcastle disease
NDV: Newcastle disease virus
NP: Nucleocapsid protein
P: Phosphoprotein
M: Matrix protein
F: Fusion protein
HN: Hemagglutinin-neuraminidase
L: RNA-dependent RNA polymerase
IG: Intergenic
