# Supplementary material for: Reverse Genetics Assembly of Newcastle Disease Virus Genome Template Using Asis-Sal-Pac BioBrick Strategy
Source: Biol Proced Online. 2020 May 1;22:9. doi: 10.1186/s12575-020-00119-3 (PMC7193399; doi:10.1186/s12575-020-00119-3)
Supplement: Supplementary file 1 — Additional file 1: Table S1. Intracerebral pathogenicity index (ICPI) for three isolated NDVs in 1 day-old chickens. [file 12575_2020_119_MOESM1_ESM.docx]

**Supplementary Table S1: Intracerebral pathogenicity index (ICPI) for three isolated NDVs in 1 day-old chickens**

|  | MG519855 | | | MG519856 | | | MG519857 | | |
| --- | --- | --- | --- | --- | --- | --- | --- | --- | --- |
| Days after inoculation | Normal | Sick | Dead | Normal | Sick | Dead | Normal | Sick | Dead |
| 1 | 7 | 1 | 2 | 3 | 2 | 5 | 4 | 3 | 3 |
| 2 | 1 | 2 | 7 | 1 | 2 | 7 | 0 | 2 | 8 |
| 3 | 0 | 0 | 10 | 0 | 0 | 10 | 0 | 0 | 10 |
| 4 | 0 | 0 | 10 | 0 | 0 | 10 | 0 | 0 | 10 |
| 5 | 0 | 0 | 10 | 0 | 0 | 10 | 0 | 0 | 10 |
| 6 | 0 | 0 | 10 | 0 | 0 | 10 | 0 | 0 | 10 |
| 7 | 0 | 0 | 10 | 0 | 0 | 10 | 0 | 0 | 10 |
| 8 | 0 | 0 | 10 | 0 | 0 | 10 | 0 | 0 | 10 |
| sum | 8 | 3 | 69 | 4 | 4 | 72 | 4 | 5 | 71 |
| Score (Factor) | 0 | 1 | 2 | 0 | 1 | 2 | 0 | 1 | 2 |
| Total | 0 | 3 | 138 | 0 | 4 | 144 | 0 | 5 | 142 |
| ICPI | 1.8 | | | 1.85 | | | 1.84 | | |
