# Supplementary material for: Reverse Genetics Assembly of Newcastle Disease Virus Genome Template Using Asis-Sal-Pac BioBrick Strategy
Source: Biol Proced Online. 2020 May 1;22:9. doi: 10.1186/s12575-020-00119-3 (PMC7193399; doi:10.1186/s12575-020-00119-3)
Supplement: Supplementary file 2 — Additional file 2: Table S2. Mean death time (MDT) for three isolated NDVs in SPF embryonated eggs. [file 12575_2020_119_MOESM2_ESM.docx]

**Supplementary Table S2: Mean death time (MDT) for three isolated NDVs in SPF embryonated eggs**

| MG519855 | Dilutions | 24 | 36 | 48 | 60 | 72 | 84 | 96 | 108 | 120 | 132 | 144 | 156 | 168 | MLD | MDT |
| --- | --- | --- | --- | --- | --- | --- | --- | --- | --- | --- | --- | --- | --- | --- | --- | --- |
|  | 10^-1^ | 0 | 5 | 0 | 0 | 0 | 0 | 0 | 0 | 0 | 0 | 0 | 0 | 0 | 8 | 52.27 |
|  | 10^-2^ | 0 | 5 | 0 | 0 | 0 | 0 | 0 | 0 | 0 | 0 | 0 | 0 | 0 |  |  |
|  | 10^-3^ | 0 | 5 | 0 | 0 | 0 | 0 | 0 | 0 | 0 | 0 | 0 | 0 | 0 |  |  |
|  | 10^-4^ | 0 | 4 | 5 | 0 | 0 | 0 | 0 | 0 | 0 | 0 | 0 | 0 | 0 |  |  |
|  | 10^-5^ | 0 | 3 | 4 | 5 | 0 | 0 | 0 | 0 | 0 | 0 | 0 | 0 | 0 |  |  |
|  | 10^-6^ | 0 | 2 | 3 | 5 | 0 | 0 | 0 | 0 | 0 | 0 | 0 | 0 | 0 |  |  |
|  | 10^-7^ | 0 | 1 | 4 | 5 | 0 | 0 | 0 | 0 | 0 | 0 | 0 | 0 | 0 |  |  |
|  | 10^-8^ | 0 | 2 | 4 | 5 | 0 | 0 | 0 | 0 | 0 | 0 | 0 | 0 | 0 |  |  |
|  | 10^-9^ | 0 | 0 | 0 | 1 | 0 | 2 | 0 | 0 | 0 | 0 | 0 | 0 | 0 |  |  |
|  | 10^-10^ | 0 | 0 | 0 | 0 | 0 | 0 | 0 | 0 | 0 | 0 | 0 | 0 | 1 |  |  |
| MG519856 | 10^-1^ | 0 | 5 | 0 | 0 | 0 | 0 | 0 | 0 | 0 | 0 | 0 | 0 | 0 | 8 | 43.5 |
|  | 10^-2^ | 0 | 5 | 0 | 0 | 0 | 0 | 0 | 0 | 0 | 0 | 0 | 0 | 0 |  |  |
|  | 10^-3^ | 0 | 5 | 0 | 0 | 0 | 0 | 0 | 0 | 0 | 0 | 0 | 0 | 0 |  |  |
|  | 10^-4^ | 0 | 4 | 5 | 0 | 0 | 0 | 0 | 0 | 0 | 0 | 0 | 0 | 0 |  |  |
|  | 10^-5^ | 0 | 4 | 5 | 0 | 0 | 0 | 0 | 0 | 0 | 0 | 0 | 0 | 0 |  |  |
|  | 10^-6^ | 0 | 4 | 5 | 0 | 0 | 0 | 0 | 0 | 0 | 0 | 0 | 0 | 0 |  |  |
|  | 10^-7^ | 0 | 3 | 5 | 0 | 0 | 0 | 0 | 0 | 0 | 0 | 0 | 0 | 0 |  |  |
|  | 10^-8^ | 0 | 3 | 5 | 0 | 0 | 0 | 0 | 0 | 0 | 0 | 0 | 0 | 0 |  |  |
|  | 10^-9^ | 0 | 0 | 0 | 1 | 0 | 2 | 0 | 0 | 0 | 0 | 0 | 0 | 0 |  |  |
|  | 10^-10^ | 0 | 0 | 0 | 0 | 0 | 0 | 0 | 0 | 0 | 0 | 0 | 0 | 1 |  |  |
| MG519857 | 10^-1^ | 0 | 5 | 0 | 0 | 0 | 0 | 0 | 0 | 0 | 0 | 0 | 0 | 0 | 8 | 50 |
|  | 10^-2^ | 0 | 5 | 0 | 0 | 0 | 0 | 0 | 0 | 0 | 0 | 0 | 0 | 0 |  |  |
|  | 10^-3^ | 0 | 5 | 0 | 0 | 0 | 0 | 0 | 0 | 0 | 0 | 0 | 0 | 0 |  |  |
|  | 10^-4^ | 0 | 4 | 5 | 0 | 0 | 0 | 0 | 0 | 0 | 0 | 0 | 0 | 0 |  |  |
|  | 10^-5^ | 0 | 3 | 4 | 5 | 0 | 0 | 0 | 0 | 0 | 0 | 0 | 0 | 0 |  |  |
|  | 10^-6^ | 0 | 2 | 3 | 5 | 0 | 0 | 0 | 0 | 0 | 0 | 0 | 0 | 0 |  |  |
|  | 10^-7^ | 0 | 1 | 4 | 5 | 0 | 0 | 0 | 0 | 0 | 0 | 0 | 0 | 0 |  |  |
|  | 10^-8^ | 0 | 3 | 4 | 5 | 0 | 0 | 0 | 0 | 0 | 0 | 0 | 0 | 0 |  |  |
|  | 10^-9^ | 0 | 0 | 0 | 1 | 0 | 2 | 0 | 0 | 0 | 0 | 0 | 0 | 0 |  |  |
|  | 10^-10^ | 0 | 0 | 0 | 0 | 0 | 0 | 0 | 0 | 0 | 0 | 0 | 0 | 1 |  |  |
