# Supplementary material for: Reverse Genetics Assembly of Newcastle Disease Virus Genome Template Using Asis-Sal-Pac BioBrick Strategy
Source: Biol Proced Online. 2020 May 1;22:9. doi: 10.1186/s12575-020-00119-3 (PMC7193399; doi:10.1186/s12575-020-00119-3)
Supplement: Supplementary file 3 — Additional file 3: Table S3. The NDV strains used in the analysis of phylogenetic tree. [file 12575_2020_119_MOESM3_ESM.docx]

**Supplementary Table S3: The NDV strains used in the analysis of phylogenetic tree**

| Genotype/Subgenotype | Country | Gene Bank NO. |  | Genotype/Subgenotype | Country | Gene Bank NO. |  |
| --- | --- | --- | --- | --- | --- | --- | --- |
| VIc | China | AF458020 | 31 | VIId | China | DQ363536 | 1 |
| VIb | Hungary | AJ880277 | 32 | VIId | China | FJ608334 | 2 |
| VIb | China | FJ865434 | 33 | VIId | China | FJ608345 | 3 |
| VIb | USA | FJ410147 | 34 | VIId | China | KC542895 | 4 |
| VIb | Argentina | AY734535 | 35 | VIId | China | AF456441 | 5 |
| VIe | China | DQ417113 | 36 | VIId | China | AY325797 | 6 |
| VIe | China | FJ766528 | 37 | VIId | China | GQ849007 | 7 |
| VIa | USA | EU477188 | 38 | VIId | China | JF340367 | 8 |
| VIa | USA | EU477192 | 39 | VIId | Iran | MG519855 | 9 |
| VIa | China | HM06342 | 40 | VIId | Iran | MG519856 | 10 |
| VIa | China | GU551934 | 41 | VIId | Iran | MG519857 | 11 |
| V | USA | AY562986 | 42 | VIId | China | KC542892 | 12 |
| V | USA | AY562990 | 43 | VIId | China | KC542903 | 13 |
| VIII | China | FJ751919 | 44 | VIIe | China | AF162714 | 14 |
| VIII | China | FJ751918 | 45 | VIIe | China | AF456437 | 15 |
| IV | China | EU293914 | 46 | VIIe | China | AF358786 | 16 |
| IV | Netherlands | AY741404 | 47 | VIIe | Japan | AB853927 | 17 |
| XI | France | HQ266602 | 48 | VIIe | China | DQ067447 | 18 |
| XI | France | HQ266603 | 49 | VIIe | China | DQ485256 | 19 |
| I | USA | AY562991 | 50 | VIIe | China | JX193075 | 20 |
| I | Australia | AY935490 | 51 | VIIf | China | AY028995 | 21 |
| II | China | JF950510 | 52 | VIIf | China | AF458010 | 22 |
| II | Germany | Y18898 | 53 | VIIg | China | EU167540 | 23 |
| III | Japan | M24700 | 54 | VI | USA | AY562988 | 24 |
| III | China | EF201805 | 55 | VIc | Japan | AB853926 | 25 |
| IX | China | AF458023 | 56 | VIc | China | AF458018 | 26 |
| IX | China | FJ436302 | 57 | VIc | China | AF458017 | 27 |
| IX | China | FJ436305 | 58 | VIc | Japan | AB465606 | 28 |
| Class I | China | FJ487637 | 59 | VIc | South Korea | GQ507801 | 29 |
| Class I | Hungary | DQ097393 | 60 | VIc | China | AF458021 | 30 |
