# Supplementary material for: Reverse Genetics Assembly of Newcastle Disease Virus Genome Template Using Asis-Sal-Pac BioBrick Strategy
Source: Biol Proced Online. 2020 May 1;22:9. doi: 10.1186/s12575-020-00119-3 (PMC7193399; doi:10.1186/s12575-020-00119-3)
Supplement: Supplementary file 4 — Additional file 4: Table S4. List of primers for identification, amplification, mutagenesis, annealing, and sequencing. [file 12575_2020_119_MOESM4_ESM.docx]

**Supplementary Table S4: List of primers for identification, amplification, mutagenesis, annealing, and sequencing**

| **Application** | **Tm** | **Primer Sequence (5'-3')** | **Name** |
| --- | --- | --- | --- |
| Identification | 63.9 | CGGAGTGTGAAAGTCATCATTCA | NDV-FF1 |
|  | 62.6 | CATTGACAAACTGCTGCATCT | NDV-FR1 |
| Identification | 65.3 | GCAACAGCGGCACAGATAAC | NDV-FF2 |
|  | 60.6 | CTTCTCCATAATTTTGCGATATGAT | NDV-FR2 |
| Identification | 66.2 | TATTCAAAGACTGAAGGCGCACTC | NDV-FF3 |
|  | 64.9 | GTTGTTGGTGATGTGGTAGAACG | NDV-FR3 |
| Mutagenesis | 80.7 | ACGC**GTCGAC**ggtggtttg**TTAATTAA**CACGGGTAGAAGAGTCTG | NDV-Fgene-F |
|  | 76.2 | ACCTATAA*GG*CG*CCC*TTGTCTCC*C*TCCTCC | NDV-FRmut |
| Mutagenesis | 76.2 | GGAGGA*G*GGAGACAA*GGG*CG*CC*TTATAGGT | NDV-FFmut |
|  | 68.1 | ATAGTTTA**GCGATCGC**TTTTTTCTTAAGTCTTCT | NDV-Fgene-R |
| Amplification | 78 | ACGC**GTCGAC**ggtggtttg**TTAATTAA**ACCAAACAGAGAATCTGT | NDV-Ngene-F |
|  | 69.3 | ATAGTTTA**GCGATCGC**TTTTTTCTAATACCTTGG | NDV-Ngene-R |
| Amplification | 78.9 | ACGC**GTCGAC**ggtggtttg**TTAATTAA**ATACGGGTAGAAGAGAGA | NDV-Pgene-F |
|  | 65.7 | ATAGTTTA**GCGATCGC**TTTTTTCTTAATTTCTAA | NDV-Pgene-R |
| Amplification | 78 | ACGC**GTCGAC**ggtggtttg**TTAATTAA**TACGGGTAGAATCAAAGT | NDV-Mgene-F |
|  | 68.1 | ATAGTTTA**GCGATCGC**TTTTTTCTAATTTGCTAG | NDV-Mgene-R |
| Amplification | 80.7 | ACGC**GTCGAC**ggtggtttg**TTAATT**AACACGGGTAGAAGAGTCTG | NDV-Fgene-F |
|  | 68.1 | ATAGTTTA**GCGATCGC**TTTTTTCTTAAGTCTTCT | NDV-Fgene-R |
| Amplification | 79.8 | ACGC**GTCGAC**ggtggtttg**TTAATTAA**CTACTGGGAACAAGCAAC | NDV-HNgene-F |
|  | 66.9 | ATAGTTTA**GCGATCGC**TTTTTTCTTAATAAAGTG | NDV-HNgene-R |
| Amplification | 77.7 | GC**GTCGAC**ggtggtttg**TGTACA**GTGCTCTTTG | NDV-L1-1-Sal-BsrG-F |
|  | 71.7 | ATAGTTTA**GCGATCGC**ACCAAACAGAGATTTGGT | NDV-Lgene-R |
| Amplification | 76.3 | GC**GTCGAC**gtcta**GCTAGC**AGATTATGCCCG | NDV-L1-2-Sal-NheI-F |
|  | 59.2 | ATAGTTTA**TGTACA**GACAGCATAAC | NDV-L1-2-BsrG-R |
| Amplification | 79.8 | ACGC**GTCGAC**ggtggtttgCC**TTAATTAA**GGTACAAAAAGCATTGAGAT | NDV-Lgene-F |
|  | 72.5 | cta**GCTAGC**GTCAGCGAGCACATATTAGAAGA | NDV-L2-NheI-R |
| Amplification | 79.8 | ACGC**GTCGAC**ggtggtttg**TTAATTAA**GATCCGGCTGCTAACAAA | NDV-T7terminator-F |
|  | 72.9 | ATAGTTTA**GCGATCGC**ATCCGGATATAGTTCCTC | NDV-T7terminator-R |
| Annealing | 96.6 | **TCGAC**ggtggtttg**TTAATT**AAGGGTCGGCATGGCATCTCCACCTCCTCGCGGTCCGACCTGGGCATCCGAAGGAGGACGTCGTCCACTCGGATGGCTAAGGGAGAGCTCGGCG**AT** | NDV-HDV-F |
|  | 96.7 | CGCCGAGCTCTCCCTTAGCCATCCGAGTGGACGACGTCCTCCTTCGGATGCCCAGGTCGGACCGCGAGGAGGTGGAGATGCCATGCCGACCCTTAATTAAcaaaccacc**G** | NDV-HDV-R |
| Annealing | 78.9 | TCGAG**GTCGAC**ggtttgggtttg**TTAATT**AA**GCGATCGC**G | NDV-pacbrick linker-F |
|  | 76.9 | AATTC**GCGATCG**C**TTAATTAA**caaacccaaaccGTCGACC | NDV-pacbrick linker-R |
| Sequencing | 52.4 | GTAAAACGACGGCCAGT | pL2-M13F |
| Sequencing | 52.4 | GTAAAACGACGGCCAGT | pLH- M13F |
| Sequencing | 52.4 | GTAAAACGACGGCCAGT | pLFmut-M13F |
| Sequencing | 52.4 | GTAAAACGACGGCCAGT | pLM-M13F |
| Sequencing | 52.4 | GTAAAACGACGGCCAGT | pLP-NF3 |
| Sequencing | 52.4 | GTAAAACGACGGCCAGT | pLN-M13F |

The linker sequences are shown in lowercase; restriction enzyme sites are shown in bold, and nucleotide substitutions for mutagenesis are underlined.
